# MyoData: An expression knowledgebase at single cell/nucleus level for the discovery of coding-noncoding RNA functional interactions in skeletal muscle

**DOI:** 10.1016/j.csbj.2021.07.020

**Published:** 2021-07-26

**Authors:** Davide Corso, Francesco Chemello, Enrico Alessio, Ilenia Urso, Giulia Ferrarese, Martina Bazzega, Chiara Romualdi, Gerolamo Lanfranchi, Gabriele Sales, Stefano Cagnin

**Affiliations:** a Department of Biology, University of Padova, Via Ugo Bassi 58/b, 35131 Padova, Italy; b CRIBI Biotechnology Centre, University of Padova, Via Ugo Bassi 58/b, 35131 Padova, Italy; c CIR-Myo Myology Center, University of Padova, Via Ugo Bassi 58/b, 35131 Padova, Italy

**Keywords:** Single myofiber, Single nucleus, Non-coding RNAs, Database, Networks, Pathways

## Abstract

Non-coding RNAs represent the largest part of transcribed mammalian genomes and prevalently exert regulatory functions. Long non-coding RNAs (lncRNAs) and microRNAs (miRNAs) can modulate the activity of each other. Skeletal muscle is the most abundant tissue in mammals. It is composed of different cell types with myofibers that represent the smallest complete contractile system. Considering that lncRNAs and miRNAs are more cell type-specific than coding RNAs, to understand their function it is imperative to evaluate their expression and action within single myofibers. In this database, we collected gene expression data for coding and non-coding genes in single myofibers and used them to produce interaction networks based on expression correlations. Since biological pathways are more informative than networks based on gene expression correlation, to understand how altered genes participate in the studied phenotype, we integrated KEGG pathways with miRNAs and lncRNAs. The database also integrates single nucleus gene expression data on skeletal muscle in different patho-physiological conditions. We demonstrated that these networks can serve as a framework from which to dissect new miRNA and lncRNA functions to experimentally validate. Some interactions included in the database have been previously experimentally validated using high throughput methods. These can be the basis for further functional studies. Using database information, we demonstrate the involvement of miR-149, -214 and let-7e in mitochondria shaping; the ability of the lncRNA Pvt1 to mitigate the action of miR-27a via sponging; and the regulatory activity of miR-214 on Sox6 and Slc16a3. The MyoData is available at https://myodata.bio.unipd.it.

## Highlights

• Regulation of gene expression through non-coding RNAs at single myofiber and nucleus resolution.
• Reinterpretation of KEGG pathways with microRNA and long non-coding RNA activities.
• miR-149, -214, and let-7e alter mitochondrial shape.
• The long non-coding RNA Pvt1 is a sponge for miR-27a.
• miR-208b regulates Sox6; miR-214 regulates both Sox6 and Slc16a3.

## Graphical abstract

**Figure:**
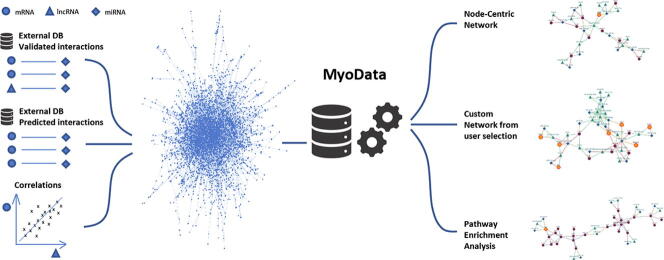

## 1 Introduction

Skeletal muscle is one of the most abundant organs in mammals as it accounts for 40–45% of the total body mass of healthy individuals. It is involved in body movement, metabolism, and protection of internal organs. Skeletal muscle is composed of different types of cells (neurons, blood cells, endothelial cells, etc.) [1] mixed with contractile myofibers, which are the tissue’s parenchymal cells and exert the previously mentioned functions. Myofibers are large, multinucleated cells that are enwrapped by connective tissue to form fasciculi [2]. Skeletal muscles from different parts of the body have distinct physiological characteristics, such as in their metabolism, contractility, elasticity, and resistance to fatigue. Distinct physiological tracts of muscles reflect specific biochemical traits of myofibers that compose each muscle. Myofiber types are plastic and respond to specific stimuli by changing their traits and thus altering the physiology of the entire muscle to which they belong. Myofibers are canonically distinguished according to the expression of the different isoforms of the myosin heavy chain (MyHC). In humans, the identified myofibers include type 1 myofibers, which are mitochondria-rich and rely on oxidative metabolism; type 2a fibers, with oxidative fast-twitch characteristics; and the glycolytic type 2x fibers [3]. In addition to the aforementioned fibers, mice have type 2b myofibers that are glycolytic fast-twitch myofibers [4]. Due to the plasticity of skeletal muscle, myofibers with mixed MyHC isoforms are also present (type 2a2x or 2x2b myofibers).

Aside from classifying myofibers by MyHC isoform content, a novel myofiber classification based on single-myofiber transcriptomic profiles was recently proposed that identifies specific transcriptional biomarkers for each myofiber type [5]. This method classifies myofibers as transcriptional slow (tS) and transcriptional intermediate (tI) with oxidative metabolism, and transcriptional fast (tF) with glycolytic metabolism. Transcriptional classification of myofibers appears to be more suitable to identify fibers in dynamic transition between different phenotypes.

Several non-coding RNAs, such as microRNAs (miRNAs), are involved in the specification of numerous muscle functions comprising development [6], pathology [7], and myofiber metabolism [5]. Not only do miRNAs participate in the regulation of muscle functions, but also long non-coding RNAs (lncRNAs) [8], [9], [10], [11]. For example, we demonstrated that lncRNAs differentially expressed in slow and fast contracting myofibers regulate myofiber metabolism [12].

Complex cellular composition, fiber diversity, and dynamic changes of fiber phenotype imply that expression patterns at the single-cell level should be used to really understand the molecular bases of skeletal muscle regulation. This level of investigation is particularly important when dealing with non-coding RNAs because this class of regulative molecules shows a stronger cell type-specific expression than coding RNAs [13], [14], [15], [16], [17]. Furthermore, it should be noted that in any differentiated cell, non-coding and coding RNAs form an intricate cross-talking network of interactions to regulate the actual gene expression patterns. As a result of these interactions, miRNAs regulate coding RNAs through post-transcriptional mechanisms [18], and lncRNAs, in turn, regulate the expression of coding RNAs [19] but also miRNA function by sponging them [20].

In this work, to better understand the molecular mechanisms involved in the functional specification of the different myofiber types, we integrated gene expression data of coding and non-coding RNAs to produce comprehensive lncRNAs-miRNAs-mRNAs interaction networks. Recently, different techniques have been developed to analyze gene expression at single-cell or single-nucleus level [21] permitting us to distinguish, at an unprecedented scale of analysis, not only how many differentially committed cells populate complex tissues but also how individual cells are affected and respond to different physio-pathological conditions [22], [23]. One limitation of this type of analysis is that they allow the detection of only polyadenylated RNAs, excluding from the analysis non-polyadenylated mature miRNAs. To overcome this problem, we integrated available single nucleus RNA-seq (snRNA-seq) analyses on skeletal muscle tissue with our previously determined networks describing single myofibers gene interactions. Gene networks based on expression correlations are known to produce inferred interactions that result as false positives after experimental validation. This approach is also less manageable and intuitive than the building of networks based on manually curated pathways. On the other hand, manually curated pathways do not consider the regulative action of miRNAs and lncRNAs. We introduced gene expression regulation based on non-coding RNAs in KEGG pathways to allow for a better description of specific changes in different myofiber types or in different studies based on snRNA-seq. We experimentally confirmed some interactions identified in our database showing the involvement of specific miRNAs in the regulation of the mitochondrial network. Moreover, we confirmed the activity of some lncRNAs as miRNA sponges and the role of some miRNAs in the regulation of genes that are known markers of myofiber specificity.

## 2 Material and Methods

### 2.1 Gene expression data and processing

Single myofiber gene expression data were collected from Gene Expression Omnibus (GEO) and Sequence Read Archive (SRA) databases using the following IDs: GSE98328, SRX2768351, SRX2768352, SRX2768353 [5], and GSE112716 [12]. For snRNA-seq, we used processed data retrieved from [24], [25], [26] as an example of muscle pathology, fiber typing, and ageing respectively. Microarray gene expression data were processed as follow. Agilent microarray mouse platform was re-annotated (Gencode annotation release vM22, evidence-based annotation of the mouse genome GRCm38, version M22 Ensembl 97) both for coding and non-coding RNAs. Microarray data were normalized using quantile normalization separately for protein-coding and long non-coding genes. The dataset includes 10 biological replicates for each myofiber type considered (1, 2A, 2A/2X, 2X, 2X/2B, and 2B). Myofibers were sub-grouped in transcriptional slow (type 1), transcriptional intermediate (type 2A, 2A/2X, and 2X), and transcriptional fast (type 2X/2B and 2B). RNA sequencing data for miRNA identification were mapped to the known mouse miRNA precursors from the miRBase database (Ver. 19) using the mapper module of miRDeep with default settings. Quantize module was used to normalize read counts of mature miRNAs.

### 2.2 Gene expression correlation

We computed the level of expression correlation among different RNA categories using the Spearman index as follows: mRNA – lncRNA; mRNA – miRNA; lncRNA – miRNA.

Correlations were obtained using the ‘cor’ function provided by the ‘stats’ library of the R language. All correlations were filtered based on specific thresholds: for the mRNA – lncRNA comparisons we required a correlation greater or equal to 0.45; for the miRNA – mRNA and miRNA – lncRNA comparisons, we selected correlations below −0.35. Furthermore, a permutational test was implemented to assess statistical significance: we computed an empirical p-value using 1,000 random permutations of the experimental measures.

### 2.3 Interactions between miRNAs and mRNAs and miRNAs and lncRNAs

We collected validated and predicted interactions from multiple sources. Specifically:

• miRNA – mRNA validated interactions were downloaded from TarBase v7.0 [27], [28] and the Encyclopedia of RNA Interactomes [29], [30] (ENCORI: HITS-CLIP validation, data downloaded November 27, 2020)
• miRNA – mRNA predicted interactions were extracted from miRDB (v6.0) [31], [32], miRmap (version of 10-Jan-2013) [33], [34], RNA22 [35] (full sets of prediction of Mus musculus based on Ensembl 96, miRBase 22 and RNA22v2), PITA [36] (both files with zero flank and with a flank of 3 and 15 bases upstream and downstream)
• miRNA – lncRNA validated interactions were downloaded from DIANA-tool (LncBased v.2) [37] and ENCORI [29], [30] (HITS-CLIP validation, data downloaded November 27, 2020).

All interactions were further filtered based on correlation results, using the same thresholds described in the previous section.

### 2.4 Functional circuits

We used the collected interactions to identify minimal functional circuits, defined as groups of three interacting nodes: one mRNA, one lncRNA, and one miRNA. We found a total of 9,625,735 circuits, divided as follows: 9,502 including validated interactions and 9,616,233 containing predicted interactions.

### 2.5 Node-Centric network

Each web page describing an mRNA, miRNA, or lncRNA displays a small network representing a selection of the functional circuits involving searched entry. As the complete network would be too large to be practically displayed, we designed a heuristic approach to identify the most relevant interactions to be included.

We collect all edges belonging to functional circuits and for each, we compute two weights as follows:

• A weight ‘w’ defined as the p-value of the correlation between the two endpoints of that edge.
• A weight ‘wpg’ (named after the fact that it will be later used to compute the PageRank importance of each node) defined as follows:

For edges obtained from circuits including predictions:

wpg=1-w

For edges obtained from circuits including validated interactions:

$$\mathrm{wpg}=1-\frac{w}{\mathrm{sf}}$$

where *sf* is the ratio between the number of edges coming from circuits obtained from predicted and from validated interactions

The scale factor *sf* was devised to balance the relative importance of circuits including predicted and validated interactions. Indeed, the former are much more numerous than the latter; if unchecked, this imbalance would risk obscuring almost completely the contribution of validated results in the final network.

Overall, this master network derived from function circuits includes 17,886 nodes and 1,243,206 edges.

The most relevant network centered at each node is then computed using the following procedure:

1) Starting from a node of interest n, we find the subgraph induced by its neighbors within a distance of two steps in the master network.
2) We compute the PageRank of each node using the ‘wpg’ weights, and we select the top 30 nodes according to this metric. We balance types of nodes in such a list: in other terms, we try to collect 10 mRNAs, 10 miRNAs, and 10 lncRNAs to provide an even representation of the different RNA species.
3) We collect circuits involving the nodes identified in the previous step giving priority to validated interactions. This step is repeated until there are no more isolated nodes
4) Step #3 does not guarantee, by itself, that the resulting network will consist of a single connected component. Since that is our final objective, we apply the following transformation until multiple components remain:
  a. We pick the smallest and the largest components.
  b. We identify the two nodes with the highest PageRank inside those.
  c. We link the nodes together by adding the edges along the shortest path connecting them to the network obtained in step #1
  d. We add one extra node for each edge along the shortest path, in order to capture, if existing, the functional circuits having such edges as one of their sides. This step is guided by a global optimization procedure aimed at reducing the total number of nodes that have to be introduced.

### 2.6 Custom network from user selection

The Custom Network section gives the user the option to provide a list of up to 30 nodes (mRNAs, miRNAs, or lncRNAs). Our system will then generate a network representing the most relevant circuits including the nodes in the user selection. The procedure we use to build this network is similar to the one developed for the single nodes, but we employed some specific optimizations to obtain a solution in real time:

1) First of all, we keep in memory the master network, the PageRank score of all the nodes and the corresponding minimum spanning tree (MST).
2) Instead of starting from the collection of circuits, we directly compute the induced subgraph defined by the user selection.
3) If multiple connected components remain, we link them by extending the network to include the shortest path identified on the MST among the highest-scoring PageRanked nodes.

Because of graphical constraints, we limit the total number of nodes in the resulting networks to 150.

### 2.7 Single-nucleus network

We integrated snRNA-seq data into our network, starting from nucleus-type specific clusters obtained from [24], [25], [26]. We collected all the genes belonging to each cluster identified in single myofibers: myonuclei (type 1, 2A, 2X, 2B, Nr4a3+, Enah+, Ampd3 + ), nuclei from satellite cells, neuromuscular junction, myotendinous junction, and myocytes. We built type-specific networks filtering functional circuits given their overlap with each group of genes and using the same procedure described in paragraph 2.5 (Node-Centric Network) to reorganize the network.

Clusters from [24] contain two nucleus categories: wild type (WT) or delta exon 51 (DEx51) of the gene encoding for dystrophin. In this case, we extended the filtering procedure to keep the circuits identified in the two subgroups separated.

### 2.8 Pathway construction

The topologies of all KEGG pathways were retrieved from the graphite package [38]. Each network was then extended to include predicted and validated interactions involving miRNAs or lncRNAs.

Starting from a set of nodes provided by the user (the query), we perform a series of hypergeometric tests to find the list of pathways significantly overlapping such query. To this end, we use the ‘hypergeom.sf’ implementation provided by the ‘scipy’ library [39] and we corrected results using the ‘fdrcorrection’ (Benjamini-Hochberg method) provided by the ‘statsmodel’ library [40].

Results of pathway enrichment analyses are displayed in a table. Each entry is linked to a detailed view of the corresponding pathway showing the following information: the nodes in common with the user query and the most relevant circuits involving protein-coding genes, lncRNAs, and miRNAs, that overlap the pathway.

### 2.9 Software implementation

All the information about expressions, correlations, and networks (nodes and topologies) is stored in a RocksDB database and accessed through the python-rocksdb library [41].

Network algorithms to compute shortest paths, minimum spanning trees, and PageRanks are implemented by the network library [42]. The web interface was built in JavaScript on top of React [43] and fontawesome icons [44]. We relied on React-Apexcharts [45] for the display of expression plots and on React-Table [46] for the generation of dynamic tables.

Finally, we extended Cytoscape-JS [47] and the layout engine cytoscape-cola [48] to render networks.

### 2.10 Primer design

Primers to amplify the genomic region containing miRNA genes and primers for qRT-PCR analyses were designed using the Primer3Plus algorithm (http://www.bioinformatics.nl/cgi-bin/primer3plus/primer3plus.cgi, Accessed on 18th of July 2021) and analyzed for dimers and secondary structure formation with OligoAnalyzer tool (Integrated DNA Technologies). Moreover, primers were tested using the in-silico PCR tool implemented in the UCSC Genome Browser. Primer sequences were reported in the Supplementary Table S1.

### 2.11 miRNA cloning

DNA regions coding for selected miRNAs were cloned in the pCMV-MiR vector (OriGene) including 200–300 bases upstream and downstream the pre-miRNA sequence.

#### 2.11.1 PCR for inserts preparation

Genomic DNA extracted from C2C12 cells was used as template for the amplification of selected miRNA genes. PCR reaction mix was prepared as following: H_2_O, 34.25 μl; PCR Buffer 10X, 5 μl; MgCl_2_ [50 mM], 4 μl; dNTPs [10 mM], 3 μl; Primer Forward [10 μM], 1 μl; Primer Reverse [10 μM], 1 μl; Taq DNA Polymerase [2 U/μl], 0.75 μl; DNA [50 ng/μl], 1 μl. PCR amplification was done in an Eppendorf thermocycler using the following program: 5 min 95° C; (30 sec 95° C; 30 sec 58-61° C; 70 sec 72° C for 45 cycles); 10 min 72° C. Amplification was verified in 1.5% agarose gel and PCR products were purified with the GenEluteTM PCR Clean-Up Kit (Sigma-Aldrich) following the manufacturer protocol.

#### 2.11.2 Plasmid and insert digestion, ligation and bacteria transformation

pCMV-MiR vector (Origene) and PCR products were digested with the same restriction enzymes in order to perform directional cloning (AscI and XhoI; New England BioLabs). Depending on the position of restriction enzymes in forward or reverse amplification primers, we were able to clone the amplicon to allow the expression of miRNA or miRNA antisense sequences (Supplementary Table S1). Restriction reactions were performed at 37° C for 90 min in the following reaction mix: H_2_O to 50 μl; Cut Smart Buffer 10x, 1 μl; AscI [10 U/μl], 1 μl; XhoI [10 U/μl], 1 μl; Plasmid/PCR Insert, 1 μg. Digestion products were purified using the GenEluteTM PCR Clean-Up Kit (Sigma-Aldrich) following the manufacturer protocol.

50 ng of plasmid were used to ligate 1:4 M quantities of PCR product as follow: H_2_O to 20 μl; T4 Ligation Buffer 10X, 2 μl; PCR amplicon 4 M with respect to the 50 ng of the vector; digested pCMV-MiR vector 50 ng; T4 DNA ligase [10 U/μl], 1 μl. The solution was incubated at 16° C overnight and then precipitated using sodium acetate and ethanol. Pellet was resuspended in 5 μl of H_2_O RNAse free.

1 μl of ligation product and 40 μl of electro-competent bacteria (*Escherichia coli* bacteria DH10B) were mixed and the solution was subjected to an electrical field of 1.8 kV in a Gene Pulser II electroporator (BioRad). Then, 360 μl of SOC medium were added and after that, the bacterial solution was incubated at 37 °C for 1 h. Bacteria were plated on solid LB medium (LB + Agar) with kanamycin [50 μg/ml] and grown at 37° C overnight. Colony PCRs were performed in order to test the presence of the insert in the plasmid using 3 μl of a liquid bacterial culture as template. The PCR products were visualized in 2% agarose gel. For each plasmid, 5 μl of one of the positive colonies were regrown in 5 ml of LB + kanamycin medium at 37° C overnight to prepare the purified plasmid. The plasmid was extracted and purified using a PureLink HiPure Plasmid Miniprep kit (Invitrogen). To test the accuracy of the pre-miRNA sequences, all plasmids have been sequenced (Sanger Sequencing, Eurofins) and compared with the mouse reference genomic sequences derived by the UCSC Genome Browser.

### 2.12 C2C12 culture and cell transfection

C2C12 myoblasts were cultured on Tissue Culture dishes (Thermo Fisher Scientific) in proliferation medium (Dulbecco’s modified Eagle’s medium (DMEM), 10% fetal bovine serum, 1 U/ml Penicillin, 100 μg/ml Streptomycin) until reaching 80% of confluence. After cell detaching with Trypsin-ethylenediaminetetraacetic acid (Thermo Fisher Scientific) 40,000 or 60,000 cells were plated on each well of Multiwell Culture plates (Thermo Fisher Scientific) using medium without antibiotic. A sterile 13 mm round coverslip was positioned on the bottom of the wells before cell seeding. Cells were co-transfected with mitoRFP and pCMV-MiR (with cloned a specific miRNA or miRNA antisense) using the Lipofectamine 2000 (Thermo Fisher Scientific) as the transfecting agent. Transfection solution was prepared by combining and incubating two solutions at room temperature for 30 min, which contained: (solution 1) 3 μl of Lipofectamine 2000, 122 μl of Opti-MEM (Thermo Fisher Scientific); (solution 2) 2 μl of mito-RFP plasmid [100 ng/ul], 2 μl of pCMV-MiR with cloned miRNA or antisense [100 ng/ul], 121 μl of Opti-MEM (Thermo Fisher Scientific). Cells with the transfection solution were grown for 24 h at 37 °C in 5% CO_2_ in a humidified incubator. After 24 h the medium was changed with a new medium containing G418 antibiotic [0.5 mg/ml] for 4 days. G418 antibiotic was used to positively select cells transfected with pCMV-MiR.

Pvt1 silencing was performed using antisense LNA GapmeRs (Exiqon) (Pvt1 1 ACCGTAGTAGAGTTAA; Pvt1 3 AGTCAACGCTTCACAT). Cells transfected with Lipofectamine 2000 and Antisense LNA GapmeR Negative Controls (Exiqon) were used as negative controls.

### 2.13 Mitochondrial network analysis

Survived cells to G418 selection were used to evaluate mitochondrial network. In fact, mitoRFP plasmid encodes for a fluorescent tag localized in the mitochondria, which is characterized by an excitation wavelength of 555 nm and an emission wavelength of 584 nm. After G418 selection, the culture medium was removed, and a first wash was carried out with 500 μl of phosphate-buffered saline (PBS). Cells were then fixed by adding 500 μl of 4% paraformaldehyde in PBS and incubated at room temperature for 15 min. Then, three washes with PBS were performed, slides on the bottom of the wells were recovered, rinsed in distilled H_2_O, and mounted on glass slide. Slides have been observed through a confocal microscope, oil immersion objectives (63x of magnification), and exciting samples with a wavelength of 555 nm. Z-stack images of samples have been acquired and used for subsequent analyses to determine the degree of mitochondrial fragmentation.

The images were analyzed with the ImageJ software, using the MitoLoc plug-in [49]. To describe mitochondrial morphology, we used the fragmentation index (F.I.) calculated as follows: V_S_ = (V_fragment_/V_total_) ∙100 and F.I. = ($\sum_{1}^{X}\mathrm{Vs}\leq 20\%)/\left(\sum_{1}^{X}Vs\right)$.

### 2.14 Electron microscopy

Transfected C2C12 cells were fixed with 2.5% glutaraldehyde in 0.1 M sodium cacodylate buffer pH 7.4 for 1 h at 4° C, post-fixed with 1% osmium tetroxide and 1% in 0.1 M sodium cacodylate buffer for 2 h at 4° C. Samples were washed three times with water and then dehydrated in a graded ethanol series and embedded in an epoxy resin (Sigma-Aldrich). Ultrathin sections (60–70 nm) were obtained with an Ultro-tome V (LKB) ultramicrotome, counterstained with uranyl acetate and lead citrate, and viewed with a Tecnai G2 (FEI) transmission electron microscope operating at 100 kV. Images were captured with a Veleta (Olympus Soft Imaging System) digital camera.

### 2.15 Overexpression of miR-27a in mouse skeletal muscle

miR-27a was overexpressed in mouse muscles as described in [5].

### 2.16 RNA extraction and qRT-PCR analysis

Trizol (Thermo Fisher Scientific) was used to extract total RNA from C2C12 cells or skeletal muscles according to the manufacturer protocol. Briefly, 500 μl of Trizol (Thermo Fisher Scientific) per well of Multiwell Culture plates or 1 ml per 30 mg of muscle were used. 1 vol of chloroform to 5 volumes of Trizol were added and vigorously mixed; then the solution was kept on ice for 15 min and then centrifuged at 4 °C at 12,000 rpm for 20 min. The upper aqueous phase was transferred in a new Eppendorf tube and RNA was precipitated using 1:1 vol of isopropanol. RNA was resuspended in H_2_O RNAse free and tested for protein and phenol contaminations at the spectrophotometer. RNA integrity was tested with the 2100 Agilent Bioanalyzer.

RNA with RIN > 7 was used for the retrotranscription according to the following protocol. 1–3 μg of total RNA were mixed with 1 μl of oligod(T) [50 μM], 0.5 ul of random primers [20 μM], 1 μl of dNTPs and H_2_O to bring the volume to 13 μl. The solution was heated to 65° C for 5 min and then killed on ice for 2 min. 4 μl of first-strand buffer 10X, 2 μl of DTT [0.1 M] and 1 μl of Superscript II (Thermo Fisher Scientific) were added to the previous solution and all incubated at 42° C for 2 h. Superscript II was inactivated incubating the mix at 70° C for 15 min.

EvaGreen molecule (Solis BioDyne) was used to perform qRT-PCR in the CFX thermocycler (BioRad) using the following PCR cycle: 15 min 95° C, (15 sec 95° C, 20 sec 60° C, 45 sec 72° C with the fluorescence reading, and 40 cycles), 3 min 72° C. Reaction mix was 6.6 μl of H_2_O, 2 μl of Master Mix 5X, 0.2 μl of primer forward [10 μM], 0.2 μl of primer reverse [10 μM], 1 μl of cDNA [10 ng/μl].

miRNA analysis was performed using the TaqMan miRNA assays (Thermo Fisher Scientific). 10 ng of total RNA were used to retrotranscribe specific miRNAs and the U6 reference gene using the miRNA reverse transcription kit (Thermo Fisher Scientific). Real-time PCR was performed on CFX thermocycler (BioRad) using the TaqMan Universal PCR Master Mix II, no UNG (Thermo Fisher Scientific) according to the manufacturer’s protocol.

### 2.17 Luciferase assay

Myoblasts were transfected with pCMV-MiR vector containing the sequence for miR-27a or −214 and 100 pg/ml of pmirGLO Dual-Luciferase miRNA Target Expression Vector (Promega) containing the target sequence or a control sequence (primers for cloning are listed in Supplementary Table S1. Cloning was performed using SacI and XbaI restriction enzymes). Assays were performed using the Dual-Luciferase Reporter Assay (Promega), measuring firefly and renilla luciferase activities with Turner Designs TD-20/20 Luminometer (DLReady). miRNA transfections were independently replicated at least three times.

## 3 Theory and calculation

MyoData includes experimental data on gene expression on single myofibers and nucleus to calculate networks centered on each mRNA, miRNA, or lncRNA whose expression was measured. These networks are computed considering the fact that i) miRNAs induce the degradation of their targets and ii) lncRNAs may function as miRNA sponges. Therefore, interactions recorded in different databases among miRNAs and lncRNAs, and miRNAs and mRNAs were further filtered using the correlation among their expression profiles. Specifically, we require that miRNAs and lncRNAs, and miRNAs and mRNAs show negatively correlated expression patterns. On the contrary, the expression correlation between mRNAs and lncRNAs should be positive.

We have developed a heuristic approach that strikes a balance between the overall number of nodes included in each network, and their relevance, defined on the basis of the strength of their interactions and the topological distance to the user query. Moreover, the procedure results in a balanced selection over the three categories of nodes (mRNAs, miRNAs, and lncRNAs).

## 4 Results and discussion

### 4.1 MyoData resource

MyoData collects expression profiles of mRNAs, miRNAs, and lncRNAs in different myofibers and gives the user information to hypothesize their function in relationship with physio- and patho-logical differences.

The database has three main search functions:

1) The user can focus on a specific mRNA, miRNA, or lncRNA.
2) Given a list of genes, the software can extract the network containing their interactions. As described in “Materials and Methods”, we limited to 30 the number of acceptable genes in order to compute the network in real-time and to display it graphically with an acceptable level of resolution.
3) As an alternative, a gene list can be used to perform a pathway enrichment analysis. Here, we employ KEGG pathways which we extended to include miRNAs and lncRNAs. These genes are usually absent in pathways but may nonetheless influence gene expression.

**Fig. 1:**
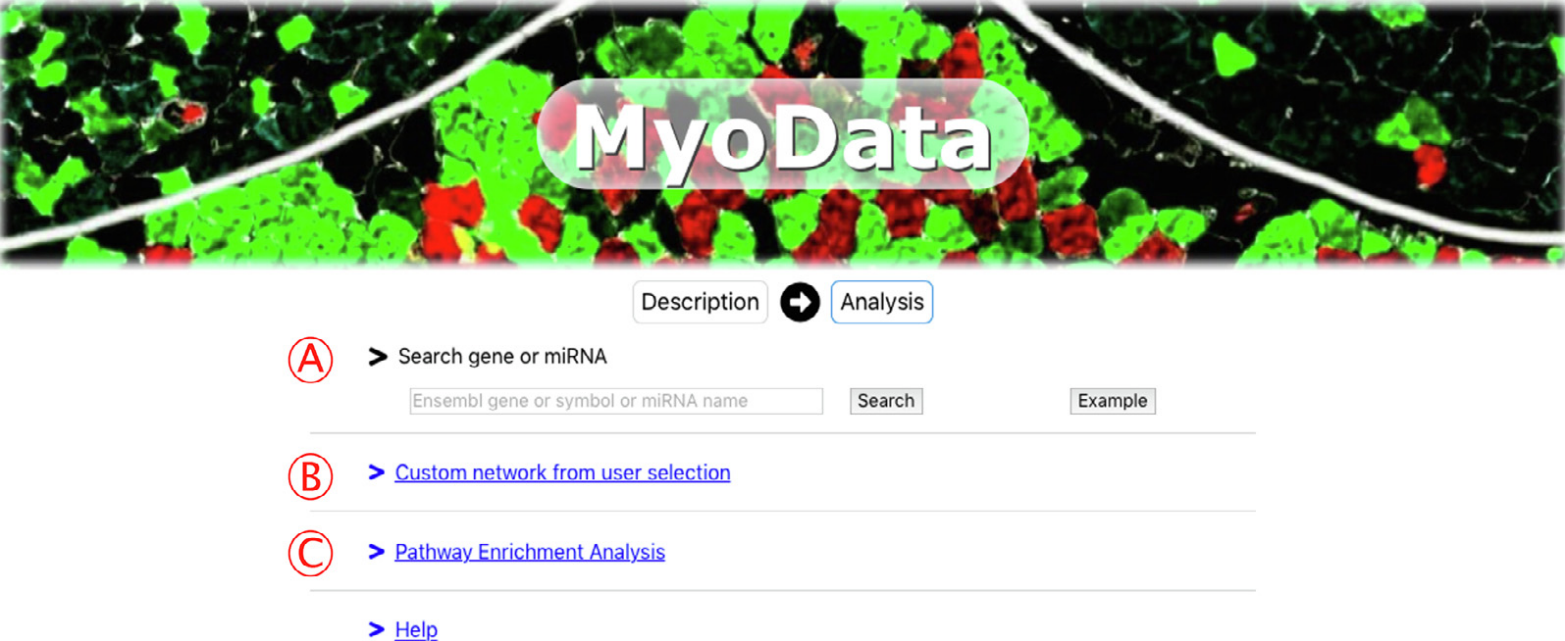
Search functions in MyoData. (A) Users can search for a single mRNA, miRNA or lncRNA. Alternatively, (B) a list of up to 30 genes can be queried to generate a network or (C) a pathway enriched with miRNA and lncRNA functions.

In all cases, MyoData accepts as an input gene symbols or ENSEMBL Gene identifiers (for mRNAs or lncRNAs) and miRNAs name (Fig. 1).

#### 4.1.1 Search for an entry: Retrieve expression on single myofibers, regulatory network centered on it

This page shows details about a single node: mRNA, miRNA, or lncRNA. It is subdivided into three sections:

1) A bar plot representing the expression values over all the available single myofibers which were experimentally assayed.
2) A network view, collecting the most relevant interactions.
3) Correlation tables.

**Fig. 2:**
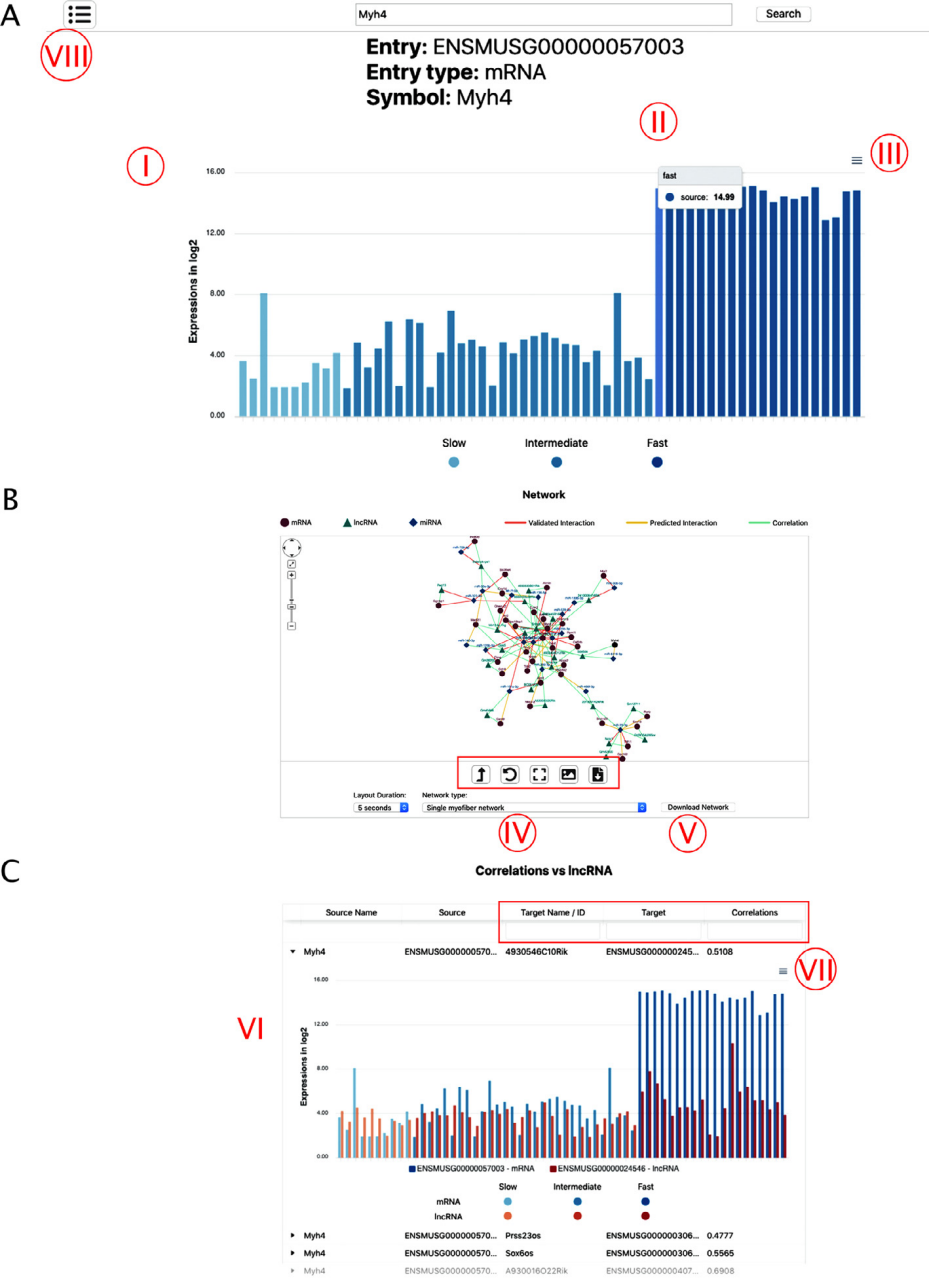
Search for a single entry. (A) Bar plot showing expression values for each biological replicate using Myh4 as an example of entry. Different gradations of blue indicate different myofiber types (I). By moving the mouse over each bar, the precise expression value appears (II). Expression tables can be downloaded (III). (B) Network visualization using Myh4 as an example of entry. Query is colored in black. Red rectangle indicates buttons to manage the network. The network can be filtered according to single nucleus RNA-seq data (IV) and can be downloaded as a table (V). (C) Correlation description using Myh4 as an example of entry. The red rectangle indicates boxes used for filtering. By clicking on the arrow next each source name, a histogram appears describing the expression of correlated genes for each sample (VI). The image can be downloaded (VII). It is possible to move to different pages by clicking the indicated button (VIII). (For interpretation of the references to colour in this figure legend, the reader is referred to the web version of this article.)

In the first section, an interactive bar plot is shown, where each expression measure is colored according to the type of myofiber it belongs to. The website also offers the possibility to download all expression tables in three different formats: svg (vector graphics), png (raster graphics), and csv (textual) (Fig. 2A).

The second section of the page displays a network collecting the interactions relevant to single myofiber types or single-nucleus cluster, which can be selected through a drop-down menu (Fig. 2B).

The layout is calculated in real-time, and the user has the option to limit the total number of seconds dedicated to this task. Moreover, different buttons give the user the possibility to further improve the layout, to reset the viewport (by centering and rescaling the network to fit the available space), to save a PNG image, or to export a tsv, or JSON file describing the network that can be later loaded into the stand-alone Cytoscape software for further analyses [50].

Network visualizations are completely interactive. By clicking on any node, its details are shown in a separate panel. Information presented includes node descriptions, Gene Ontology annotations, and external references. Similarly, edges are annotated with their correlation index, the name of the database from which they were derived the type of interactions they represent.

The third section of the page consists of a series of tables collecting the correlations computed between the selected entry and the other nodes in the database belonging to different RNA species. The user has the option to further filter the tables by searching for specific need IDs (miRBase IDs coming from miRBase v22 GRCm38). Partial matches are automatically handled: for instance, the substring “let-7a” would automatically match the full form “mmu-let-7a”. Each table row can be dynamically expanded, by clicking on a button, to display graphically the expression profiles of the two correlated nodes (Fig. 2C).

#### 4.1.2 Regulatory network from multiple entries searching

**Fig. 3:**
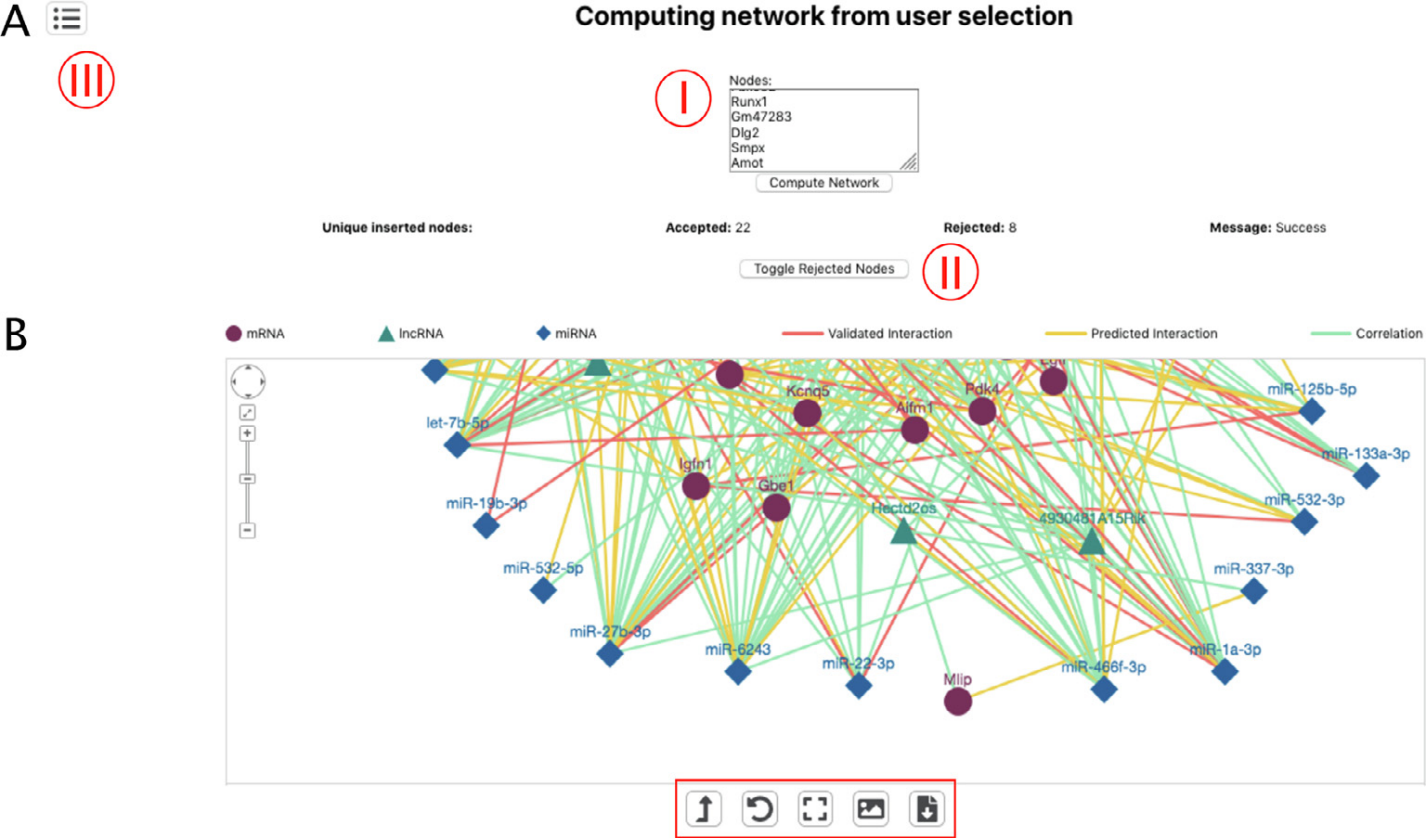
Interaction network from a list of genes. (A) Page structure for a multiple query. A list of up to 30 genes can be pasted in the box (I). After clicking on the “Compute Network” button the network will be calculated. Rejected genes can be visualized by clicking on the “Toggle rejected nodes” button (II). (B) Resulted network. The red rectangle indicates buttons to manage the network. It is possible to move to different pages by clicking the indicated button (III). (For interpretation of the references to colour in this figure legend, the reader is referred to the web version of this article.)

This page gives the user the ability to use a list of gene identifiers as a query. As described in the “Materials and Methods” section, we filter out those entries that are not present in our master network. The user has the option to display the list of such rejected IDs (Fig. 3A).

After filtering, a network is computed and displayed. The graphical format is similar to that described in the previous section, with the only difference that the layout computation can be performed for longer periods of time (up to 30 s; Fig. 3B).

#### 4.1.3 Pathway enrichment analysis

MyoData is implemented to perform a pathway enrichment analysis. This will result in the display of a table including the titles of significantly enriched pathways, their dimension, the size of the intersections with user-provided nodes, and finally the adjusted p-values for the statistical tests.

**Fig. 4:**
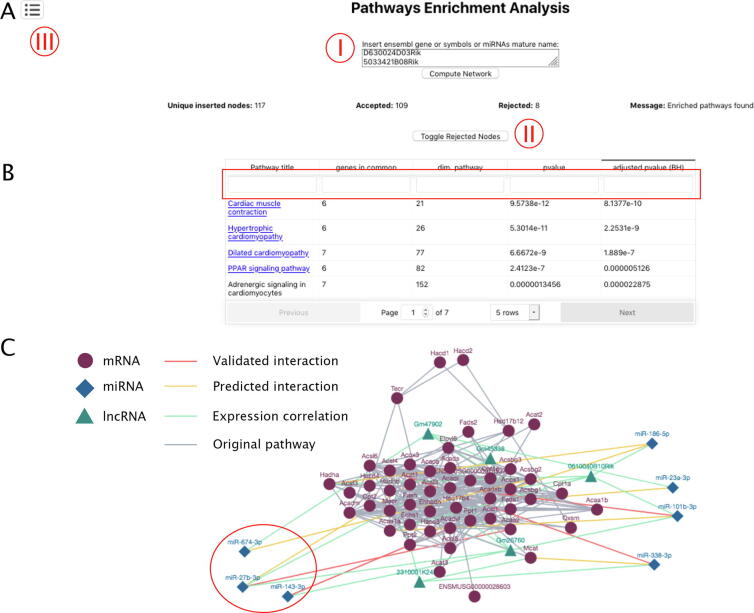
Pathways enrichment analysis. (A) Query page. In the box (I) the user can paste gene symbols and by clicking on the “Compute Network” button pathways the enrichment will be calculated. Rejected genes can be visualized by clicking on the “Toggle Rejected Nodes” button (II). (B) Results appear in a table that can be filtered according to the name of the pathway (Pathway title), number of genes identified in the pathway (genes in common), the dimension of the pathway (dim. Pathway), and statistics (pvalue and adjusted pvalue) (red rectangle). It is possible to move to different pages by clicking the indicated button (III). (C) Fatty acids metabolism pathway extended with non-coding RNAs involved in the regulation of the considered genes. Red circle indicates miRNAs discussed in the text. (For interpretation of the references to colour in this figure legend, the reader is referred to the web version of this article.)

By clicking on each pathway title, MyoData will switch to the visualization of the pathway topology, extended with the most important functional circuits (miRNA-lncRNA-mRNA interactions; Fig. 4A and B).

As an example, in Fig. 4 we used single nucleus RNA-seq results from a previous study [25]. We used genes significantly upregulated in the cluster of nuclei specific for the slow contracting myofibers (type 1 or myosin heavy chain 7; Myh7). Most enriched pathways correctly describe heart functions and the Ppar pathway (Fig. 4B). It is known that slow myofibers have isoforms of contractile proteins similar to heart, and peroxisome proliferator-activated receptor δ (PPARδ) induces a switch to form increased numbers of type 1 myofibers [51]. Prevalently, the metabolism of type 1 myofibers is based on lipid oxidation [5] and the “Fatty acids metabolism” is one of the most enriched pathways (Supplementary Table S2). Interestingly in the pathway corresponding to “Fatty acid metabolism” among other miRNAs we identified miR-27b that is considered a hub in the lipid metabolism [52], miR-674, which is associated with circulating lipids [53], and miR-143, which is already known to regulate lipid metabolism [54] (Fig. 4C).

### 4.2 Data validations

To demonstrate the potentiality and the validity of data extracted from the MyoData resource, we performed four case studies focused on important aspects of the skeletal muscle physiology: i) the modulation of mitochondrial shape by miRNAs that may impact muscle metabolism; ii) the action of lncRNAs as miRNA sponges; iii) the co-participation of different non-coding RNAs in the regulation of myofiber functions; iv) the improvement of snRNA-seq information.

#### 4.2.1 Case study 1: Identification of miRNAs impacting on the mitochondrial shape

**Fig. 5:**
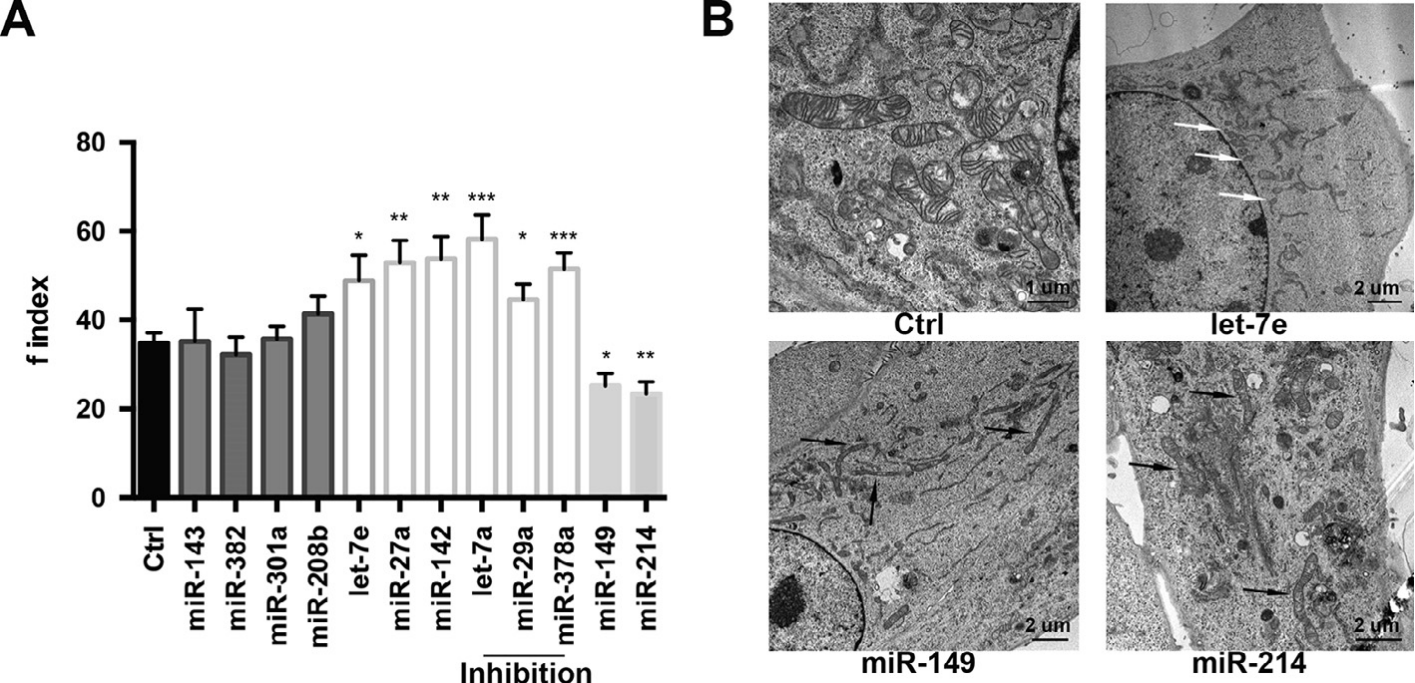
miRNAs regulate mitochondrial shape. (A) Quantification of the fragmentation index, f index, of mitochondrial networks after miRNA transfections; n = at least 20 mitochondria for each condition (three independent transfections per each miRNA). Dark grey bars represent f index associated with miRNAs not affecting mitochondrial shape; white bars represent f index associated with miRNAs that induce mitochondrial fragmentation; light grey bars represent f index associated with miRNAs that induce mitochondria fusion. Among miRNAs inducing mitochondrial fragmentation those that were inhibited are indicated. Significance was calculated using *t*-test between control and each treated sample considering unequal variance between samples. * P ≤ 0.05, ** P ≤ 0.005, *** P ≤ 0.0005. Indicated statistical significance is referred to the control (Ctrl). Error bars represent SEM. (B) Electron microscopy of C2C12 cells transfected with pCMV-MiR vectors to upregulate specific miRNAs. Black arrows indicate elongated mitochondria in cells overexpressing miR-149 and −214; white arrows indicate fragmented mitochondria in cells overexpressing let-7e.

MyoData outputs the expression of miRNAs in different myofiber types permitting users to hypothesize their function based on physiological differences of myofibers. For example, by searching for miR-214, −142, −208b, −382, and let-7e in the MyoData, users will see that these miRNAs are not expressed in intermediate myofibers, the most oxidative ones [5]. The modulation of these miRNAs likely impacts the expression of proteins controlling metabolism in this type of muscle cells. As proof of principle, we tested this hypothesis by evaluating mitochondrial shape, which is a readily measurable phenotype and is important for skeletal muscle metabolism and functions [55], [56], [57], [58]. In addition to the aforementioned miRNAs, we also included miR-301a, −29a, −143, −27a, −149, −378a, and let-7a because they target several genes coding for mitochondrial proteins (Supplementary Table S3). We tested the inhibition of miR-378a using antisense sequences since miR-378a knock-down was previously shown to induce the accumulation of abnormal mitochondria and apoptosis [59]. We confirmed that its inhibition indeed induced mitochondrial fragmentation, which is known to be a marker of apoptosis [60] (Fig. 5A). We obtained comparable results after the inhibition of miR-29a and let-7a confirming previous observations obtained in the heart [61] and HT29 cells [62]. However, the upregulation of miR-143, −382, −301a, and −208b did not change the conformation of the mitochondrial network (Fig. 5A). miR-208b is a miRNA highly expressed in slow myofibers and is involved in the specification of those types of myofibers via its blocking of Sox6 [63]. Slow oxidative myofibers are very rich in mitochondria, which correlates well with our experiments that show that the upregulation of miR-208b did not affect the mitochondrial network. miR-143 is particularly expressed in skeletal muscle and is associated with the maintenance of the satellite cell population and with aging [64], [65], similar to miR-382 [66]. Both miR-301a and −143 are upregulated in mice fed with high-fat diet [67] which impacts mitochondrial function but, according to our validation experiments, their upregulation did not affect mitochondrial conformation. The upregulation of the other tested miRNAs caused mitochondrial fission (miR-27a, −142, and let-7e) or fusion (miR-149, −214) (Fig. 5A). In summary, we confirmed that 8 out of 12 tested miRNAs altered mitochondrial shape, thereby potentially impacting the regulation of muscle metabolism. These results can be important starting points for researchers interested in studying the metabolic impact of tested miRNAs.

To confirm our previously described results, we also checked mitochondrial ultrastructure by electron microscopy. We previously showed the change in mitochondrial ultrastructure after the upregulation of miR-27a and −142 [5], therefore we tested let-7e, which according to the analysis of the f-index causes mitochondrial fission, and miR-149 and −214, which cause mitochondrial fusion, confirming in all cases previously described results (Fig. 5B).

#### 4.2.2 Case study 2: lncRNA Pvt1 as a miRNA sponge

In the MyoData database, we integrated information on miRNA–mRNA and miRNA–lncRNA interactions. This allows for the identification of miRNA–mRNA–lncRNA network triangles that describe the miRNA sponge activity of lncRNAs. We used this information to experimentally validate the activity of the lncRNA plasmacytoma variant 1 (Pvt1) as a sponge for miR-27a. We previously demonstrated that Pvt1 is involved in muscle atrophy by regulating cMYC [12]. This is possible thanks to the cytoplasmic localization of Pvt1 [12] where it acts as a sponge for miR-200 family, miR-199a, −152, and −30a in different cancers [68], [69], [70], [71].

**Fig. 6:**
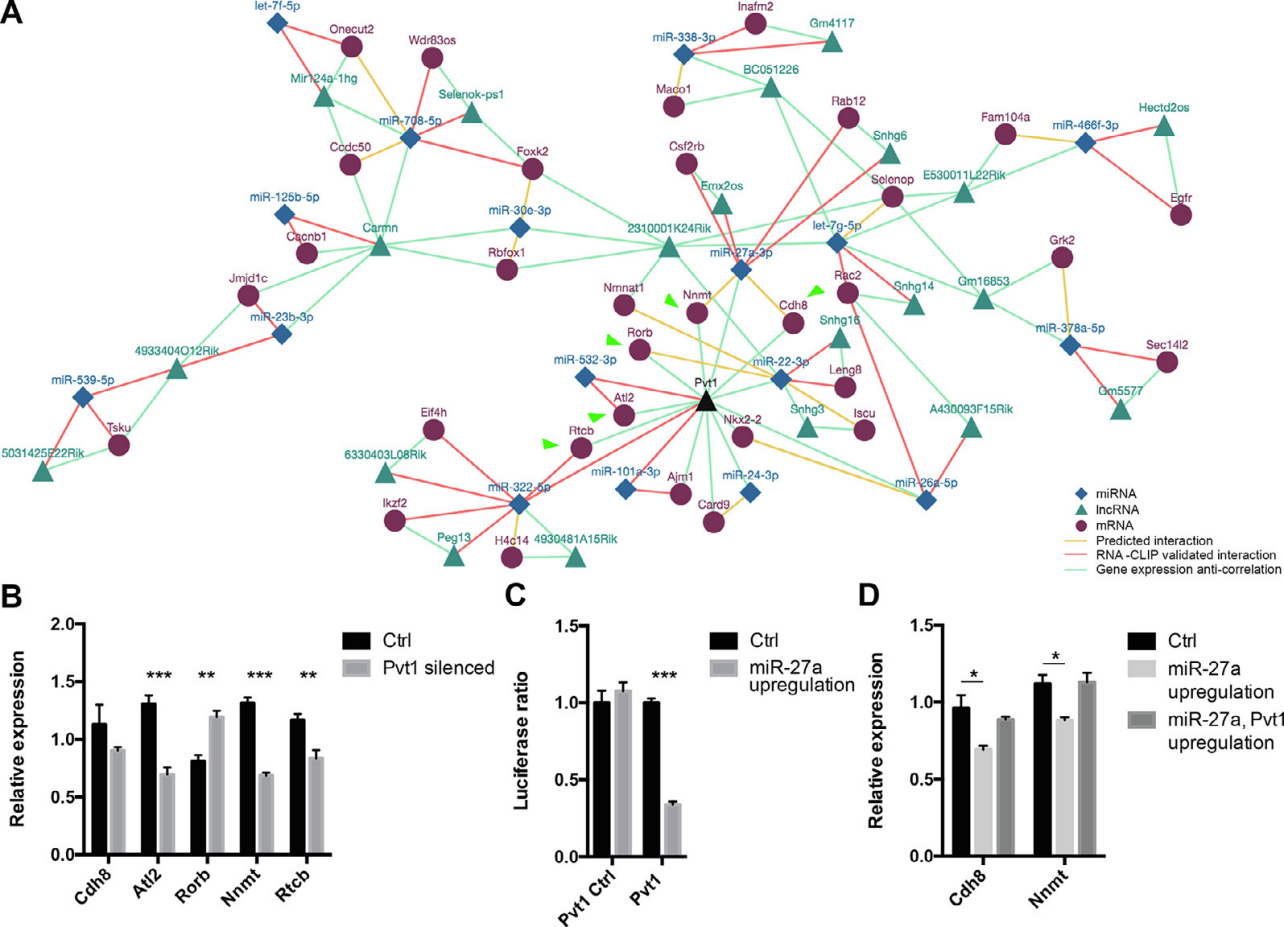
Pvt1 network. (A) Network associated to Pvt1 (black triangle) and single myofiber expression. Green arrows indicate mRNAs tested for their expression after the downregulation of Pvt1. (B) Histograms represent expression values relative to the average expression of the gene among samples. Tbp was used as control gene. At least four independent experiments were performed. Error bars indicate SEM. (C) Luciferase assays were performed to demonstrate the direct interaction between Pvt1 and miR-27a. Part of Pvt1 sequence containing the miRNA putative interaction site (or not containing; Pvt1 Ctrl) was cloned into pmirGLO vector. Firefly luciferase (reporter gene) and Renilla luciferase (control reporter for normalization) activities were measured after the transfection in C2C12 cells together with pCMV-MiR coding for miR-27a or empty pCMV-MiR (Ctrl). Data are expressed as the mean of at least five independent transfections. Error bars indicate SEM. (D) Histograms represent expression values relative to the average expression of the gene among samples. Tbp was used as control gene. Co-transfecting cells with pCMV-MiR vector coding for miR-27a and pmirGLO coding for the sequence part of Pvt1 with binding sites for miR-27a, Cdh8 and Nnmt expression were not affected. At least four biological replicates were performed. Error bars indicate SEM. For this entire figure, significance was calculated using *t*-test between control and each treated sample considering unequal variance between samples. * P ≤ 0.05, ** P ≤ 0.01, *** P ≤ 0.0005. (For interpretation of the references to colour in this figure legend, the reader is referred to the web version of this article.)

The network associated with Pvt1 outputted from MyoData identifies Pvt1 as a central node regulating miR-101a, -22, -24, -26a, -27a, -322, and -532 (Fig. 6A). Network triangles Pvt1–miR-322–Rtcb, Pvt1–miR-532–Atl2, and Pvt1–miR-101–Ajm1 have been previously experimentally validated using the HITS-CLIP technique in C2C12 cells (see edges in MyoData). To demonstrate if Pvt1 is able to act as a sponge for miR-22, −27a, -322, and -532, we evaluated the expression of the miRNA targets after Pvt1 silencing in C2C12 myotubes. We expected that the reduction of Pvt1 allows the release of miRNAs from the lncRNA, thereby permitting them to downregulate their targets. We showed that all considered targets were downregulated with the exception of RAR Related Orphan Receptor B (Rorb) that was upregulated (Fig. 6B). These data support the sponge activity of Pvt1 and its interaction with miR-532 and –322, whose relationship was derived from RNA-CLIP experiments (Fig. 6A), but do not demonstrate the direct interaction between Pvt1 and miR-27 or –22. We excluded miR-22 from experiments to validate Pvt1 interactions with miRNAs, since the miRNA target transcript Rorb was not downregulated in Pvt1-silenced cells. In the validation experiments carried out with luciferase assays, we were able to demonstrate the direct interaction of Pvt1 with miR-27a (Fig. 6C). To strengthen this result, we overexpressed miR-27a in C2C12 cells showing the downregulation of both Nnmt and Cdh8 genes. Nnmt and Cdh8 downregulation was instead attenuated in cells overexpressing the region of Pvt1 containing binding sites for miR-27a (Fig. 6D).

#### 4.2.3 Case study 3: The identification of miRNAs involved in myofiber type specification

MyoData allows for parallel searching for multiple entries. This may be useful, for example, to search if specific miRNAs influence the activity of genes coding for proteins that participate in the same cellular process or if they modulate the activity of co-regulated genes. We decided to use the database to evaluate if miR-206, −208b, and miR-214 can regulate genes involved in myofiber type specification. It was previously shown that loss of miR-214 expression in Zebrafish leads to a reduction of slow myofibers through the regulation of Su(fu) gene that participates in Hedgehog signaling. Su(fu) inhibition induces an increase in the number of slow myofibers [72]. miR-206 is predicted to regulate the expression of transcriptional repressors of the slow myosin heavy chain, such as Sox6, Purβ, and Sp3 [73].

**Fig. 7:**
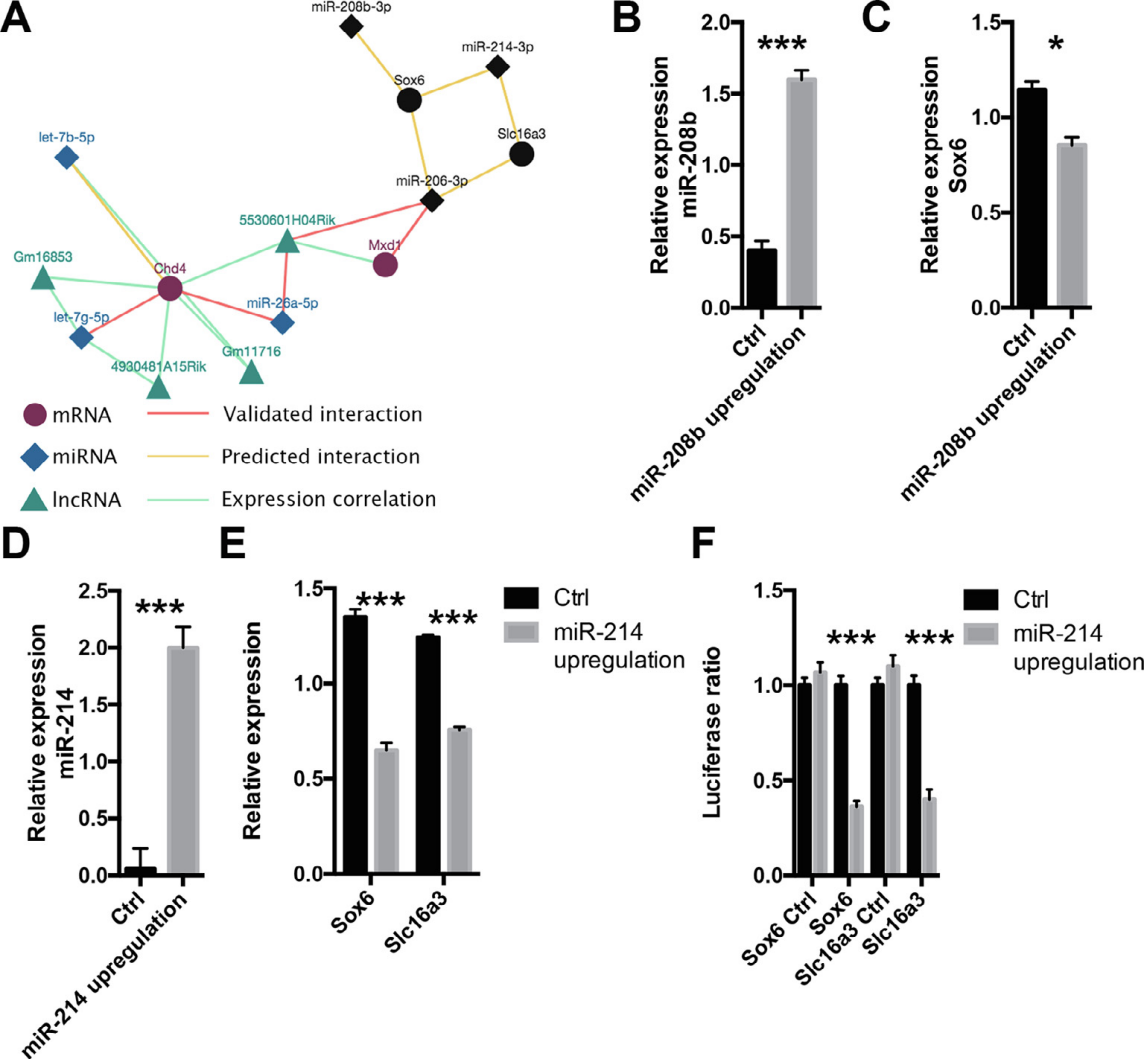
Regulation of genes that present a myofiber type dependent expression. (A) Network resulted from multiple searching of mmu-miR-206-3p, −208b-3p, −214-3p, Sox6, and Slc16a3. Black nodes indicate user nodes. (B) Histograms represent expression values relative to the average expression of the gene among samples. U6 was used as control gene. Four biological replicates were performed. Error bars indicate SEM. (C) Histograms represent expression values relative to the average expression of the gene among samples. Txn1 was used as control gene. Four biological replicates were performed. Error bars indicate SEM. (D) Histograms represent expression values relative to the average expression of the gene among samples. U6 was used as control gene. Four biological replicates were performed. Error bars indicate SEM. (E) Histograms represent expression values relative to the average expression of the gene among samples. Txn1 was used as control gene. Four biological replicates were performed. Error bars indicate SEM. (F) Luciferase assays were performed to demonstrate the direct interaction between miR-214 and Sox6 and miR-214 and Slc16a3. Part of Sox6 and Slc16a3 sequences containing the miRNA putative interaction sites (or not containing; Sox6 Ctrl and Slc16a3 Ctrl) were cloned in pmirGLO vector. Firefly luciferase (reporter gene) and Renilla luciferase (control reporter for normalization) activities were measured after the transfection in C2C12 cells together with pCMV-MiR coding for miR-214 or empty pCMV-MiR (Ctrl). Data are expressed as the mean of at least four independent transfections. Error bars indicate SEM. For this entire figure, significance was calculated using *t*-test between control and treated samples considering unequal variance between samples. * P ≤ 0.05, ** P ≤ 0.01, *** P ≤ 0.0005.

By querying MyoData for miR-206, −208b, −214, Sox6, and Slc16a3 we retrieved the network described in Fig. 7A. The three miRNAs were selected because they are exclusively expressed in slow contracting myofibers [5] and probably impact specific functions in these myofibers. Sox6 was previously reported as an important transcription factor involved in the regulation of slow myosin heavy chain gene [74], while Slc16a3 (MCT3-M/MCT4), which codifies for a lactate transporter, may be involved in the metabolism of specific myofibers. In fact, it is much more abundantly expressed in fast-twitch oxidative and fast-twitch glycolytic muscles than in slow-twitch oxidative muscles [75]. To validate this network and the suggested interactions between miRNAs and targets, we upregulated the expression of miR-208b or −214 in the C2C12 muscle cell line. In cells overexpressing miR-208b we found a clear downregulation of Sox6 (Fig. 7B and C), confirming previous evidence of this specific interaction [76]. Moreover, in C2C12 cells overexpressing miR-214, both Sox6 and Slc16a3 genes were downregulated (Fig. 7D and E). We confirmed the interaction between miR-214 and Sox6 and miR-214 and Slc16a3 via the luciferase assay (Fig. 7F) supporting the ability of miR-214 to regulate both Sox6 and Slc16a3 and its involvement in the modulation of slow myofiber functions.

#### 4.2.4 Case study 4: Integration of single nucleus and single myofiber data: New perspectives to understand Spinal and bulbar muscular atrophy

**Fig. 8:**
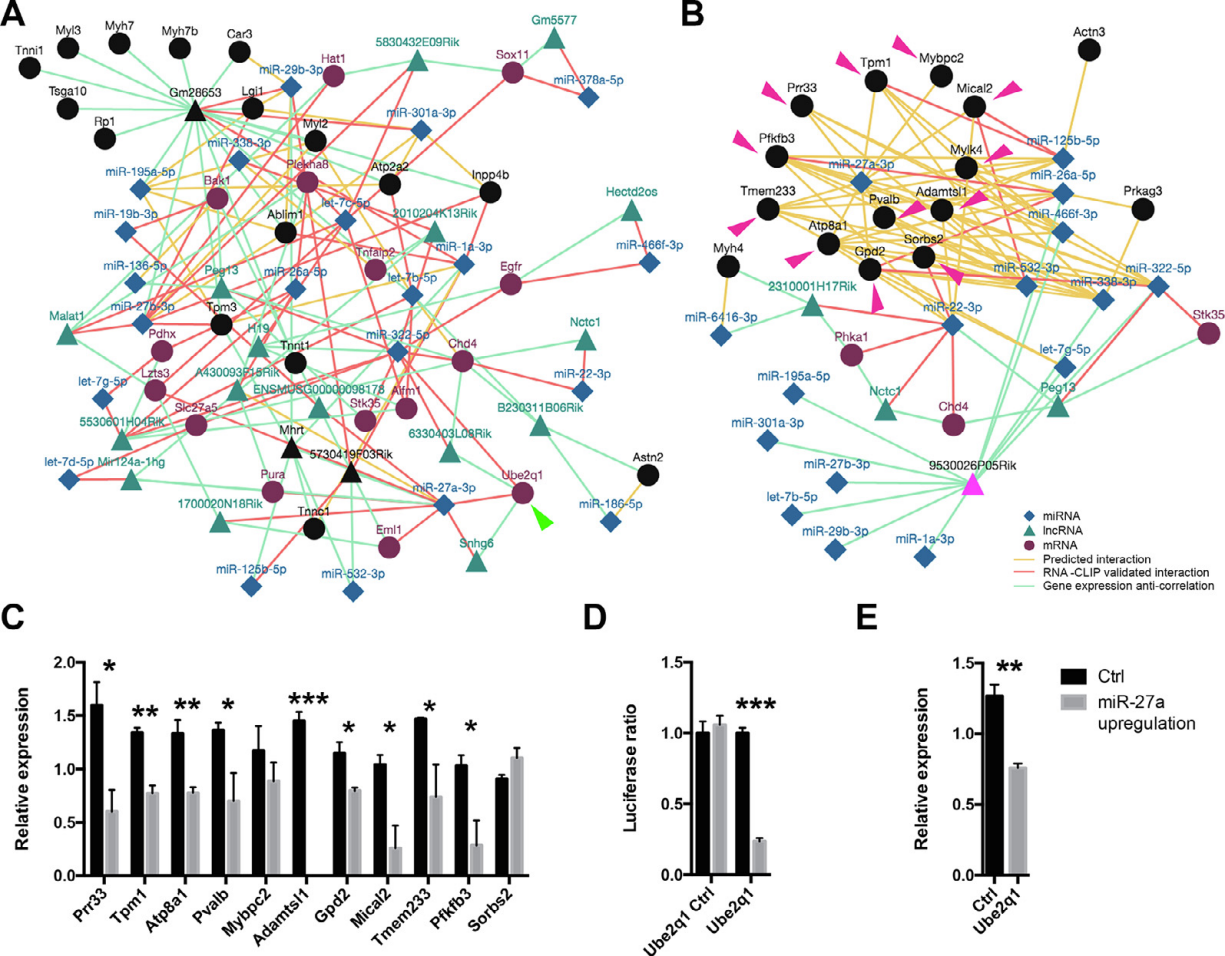
Integration of single nucleus and single myofiber data. (A) Network resulted from the interrogation of MyoData with markers from a previous study [25] for slow myofibers. Green arrow indicates Ube2q1 gene. (B) Network resulted from the interrogation of MyoData with markers from a previous study [25] for fast myofibers. Pink arrows indicate predicted targets for miR-27a. The legend is for both part A and B of the figure. Black nodes in the networks indicate searched entries from the user. (C) Gene expression of predicted targets of miR-27a shown in part B of this figure. Histograms represent expression values relative to the average expression of the gene among samples. Txn1 was used as control gene. Four biological replicates were performed. Error bars indicate SEM. (D) Luciferase assays were performed to demonstrate the direct interaction between miR-27a and Ube2q1. Part of Ube2q1 sequence containing the miRNA putative interaction sites (or not containing; Ube2q1 Ctrl) were cloned in pmirGLO vector. Firefly luciferase (reporter gene) and Renilla luciferase (control reporter for normalization) activities were measured after the transfection in C2C12 cells together with pCMV-MiR coding for miR-27a or empty pCMV-MiR (Ctrl). Data are expressed as the mean of four independent transfections. Error bars indicate SEM. (E) Relative expression of Ube2q1. Histograms represent expression values relative to the average expression of the gene among samples. Txn1 was used as control gene. Four biological replicates were performed. Error bars indicate SEM. For this entire figure, significance was calculated using *t*-test between control and treated samples considering unequal variance between samples. * P ≤ 0.05, ** P ≤ 0.01, *** P ≤ 0.0005. (For interpretation of the references to colour in this figure legend, the reader is referred to the web version of this article.)

Spinal and bulbar muscular atrophy (SBMA) is characterized by loss of motor neurons and sensory neurons, accompanied by atrophy of muscle fibers. This causes a glycolytic-to-oxidative fiber-type switch in fast-contracting skeletal muscles without a reduction of muscle mass in slow-contracting muscles (oxidative myofibers). Fast contracting muscles are also associated with a reduction of tetanic force while slow contracting muscles are not affected [77]. These observations suggest that oxidative myofibers are protected from the atrophy induced in SBMA patients. To better understand if non-coding RNAs participate in this protective mechanism we interrogated the MyoData database using the list of gene markers for slow nuclei described in [25]. The computed network is represented in Fig. 8A. Interestingly, a putative interaction of miR-27a with E2-ubiquitin ligase Ube2q1 is described. The miR-27a and Ube2q1 couple is an interesting target because we previously showed that the upregulation of miR-27a induces the increase of oxidative myofibers [5] that may have a protective role in SBMA. miR-27a is expressed only in oxidative myofibers and silent in glycolytic myofibers [5], which are the most affected in muscles of SBMA patients. First, we asked if miR-27a can modulate marker genes for fast myofibers identified by snRNA-seq [25]. To respond to this question, we evaluated the network generated by gene markers of fast myofibers. Interestingly, miR-27a was predicted to regulate 55% (11 out of 20) of fast myonuclei markers (Fig. 8 B and Supplemental Table S4). In muscles overexpressing miR-27a we confirmed by qRT-PCR that ~ 82% (9 of 11 tested genes) of genes targeted by miR-27a were downregulated (Fig. 8C). This confirms the ability of miR-27a to inhibit the fast myofiber phenotype. We then experimentally validated the suggested interaction of miR-27a with Ube2q1 using the luciferase assay (Fig. 8D). Furthermore, we showed that following the upregulation of miR-27a in muscle cells, the expression of Ube2q1 significantly decreased (Fig. 8E). In summary, our experimental data support the ability of miR-27a to modulate genes specifically expressed in fast myofibers and to buffer the expression of Ube2q1 in oxidative myofibers but not in glycolytic myofibers. This evidence may be particularly important to modulate atrophic processes in SBMA muscle. Alternatively, the upregulation of Ube2q1 in SBMA muscles [78] may be associated with the inability of myoblasts to produce new myotubes in degenerating SBMA muscles. In fact, the upregulation of Ube2q1 is associated with enhanced cell proliferation in hepatocellular carcinoma [79]. To produce myotubes, myoblasts have to withdraw from the cell cycle to fuse with each other. If withdrawal is prevented, myotubes cannot form. Finally, it is interesting to notice that Rocchi et al [77] described that a high-fat diet (HFD) ameliorates the phenotype of SBMA model mice. We showed that HFD induces the expression of miR-27a more in glycolytic than in oxidative muscles [5]. These two lines of evidence support the importance of miR-27a in SBMA and show how the database can be used to evaluate the impact of non-coding RNAs in the regulation of marker genes for specific myofibers identified by snRNA-seq experiments.

## 5 Conclusions

Gene regulation is a complex process where regulatory elements and their targets participate to form highly complex interactions thus affecting biological processes. Transcription factors (TFs) are the most well-known molecules involved in this process and several tools and databases have been published to evaluate TFs involved in the regulation of commonly altered genes [80], [81], [82], [83], [84], [85]. Some databases have been already developed to explore the gene expression of skeletal muscle [86], [87], [88], [89] and skeletal muscle after exercise [90], [91] without considering the importance of non-coding RNAs in the post-transcriptional regulation of gene expression. Different databases integrate TFs and miRNAs to describe feed-forward regulatory circuits [92], [93], [94]. Improvements in RNA sequencing technologies have allowed for the identification of single cell and single nucleus gene expression and the consequent development of several web interfaces to query mRNA and lncRNA gene expression [95], [96], [97], [98], [99], [100]. However, the integration of miRNA–lncRNA–mRNA networks at the single cell level has not been demonstrated. Such integration represents an important improvement in the comprehension of gene regulation by allowing for the identification of cell type-specific expression (higher than coding genes) and the ability of lncRNAs to sponge miRNAs.

We took advantage of our genome-wide experiments on single myofibers to implement a database to describe hypothetical and experimentally-validated interactions among miRNAs, lncRNAs, and coding RNAs to dissect gene regulation in different myofiber types. The database can be used to evaluate the impact of a single gene or group of genes (both coding and non-coding genes) on the regulation of related genes (co-expressed or coding for proteins involved in a specific pathway). MyoData integrates miRNAs and lncRNAs in KEGG pathways thereby incorporating the information of the regulation of biological processes. Mature myofibers are derived from the fusion of multiple satellite cells and are therefore a syncytium containing hundreds of nuclei that can participate differently to the cumulative gene expression [25]. We therefore included the possibility of visualizing how the networks calculated in the database change considering clusters of nuclei based on their expression retrieved from snRNA-seq experiments. We integrated these clusters with our information on miRNA expression in single myofibers because it is not feasible with current techniques to recover mature miRNA expression from snRNA-seq. We showed that this approach may be useful to identify miRNAs that regulate coding genes involved in muscle atrophy. By evaluating specific miRNAs and lncRNAs, we experimentally demonstrated that the database can guide the discovery of novel functions of non-coding RNAs in skeletal muscle. Moreover, we showed that MyoData is a valuable resource to integrate single myofiber and single nucleus gene expression information to investigate at a deeper level the molecular bases and regulations of physiology and pathology of such an abundant and complex organ as skeletal muscle.

## Declaration of Competing Interest

The authors declare that they have no known competing financial interests or personal relationships that could have appeared to influence the work reported in this paper.
